# Supplementary material for: Computed Tomography-Based Quantitative Texture Analysis and Gut Microbial Community Signatures Predict Survival in Non-Small Cell Lung Cancer
Source: Cancers (Basel). 2023 Oct 21;15(20):5091. doi: 10.3390/cancers15205091 (PMC10605408; doi:10.3390/cancers15205091)
Supplement: Supplementary file 1 [file cancers-15-05091-s001.zip › Supplementary Materials.pdf]

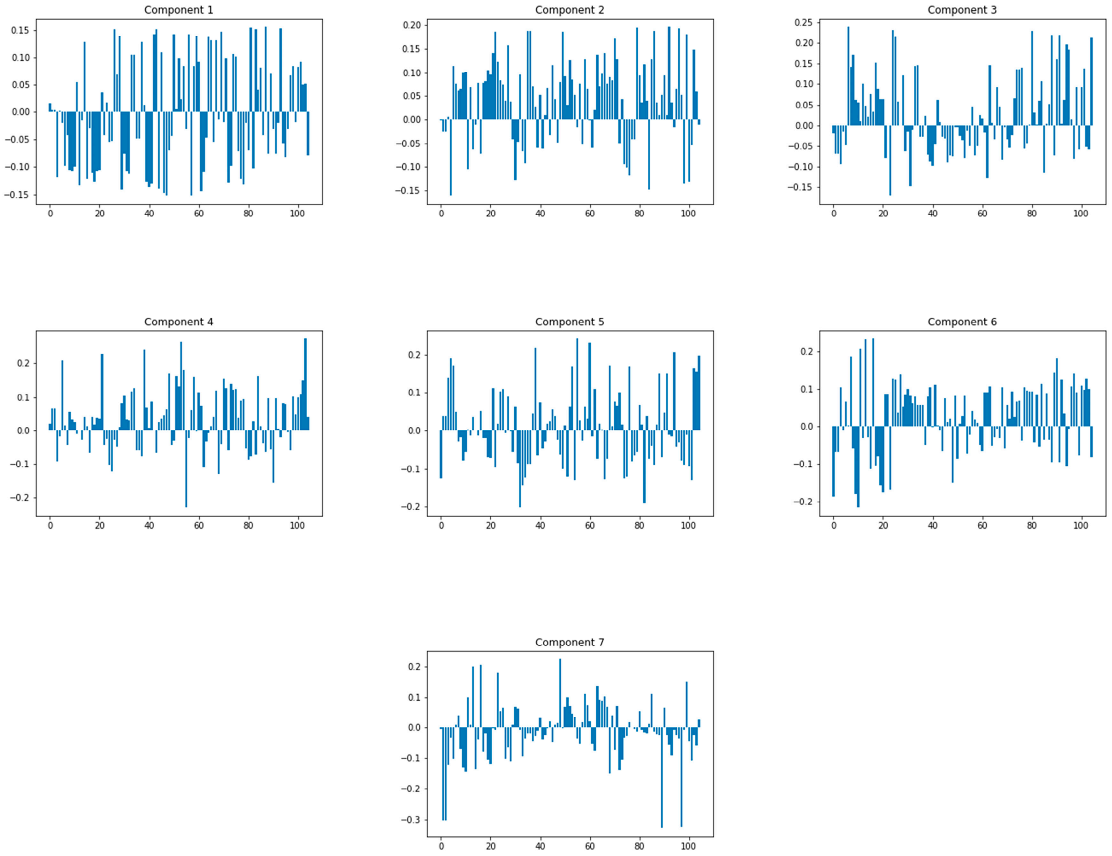

Supplementary Figure S1. Contribution of the 105 QTA features to Principal Components generated by PCA.

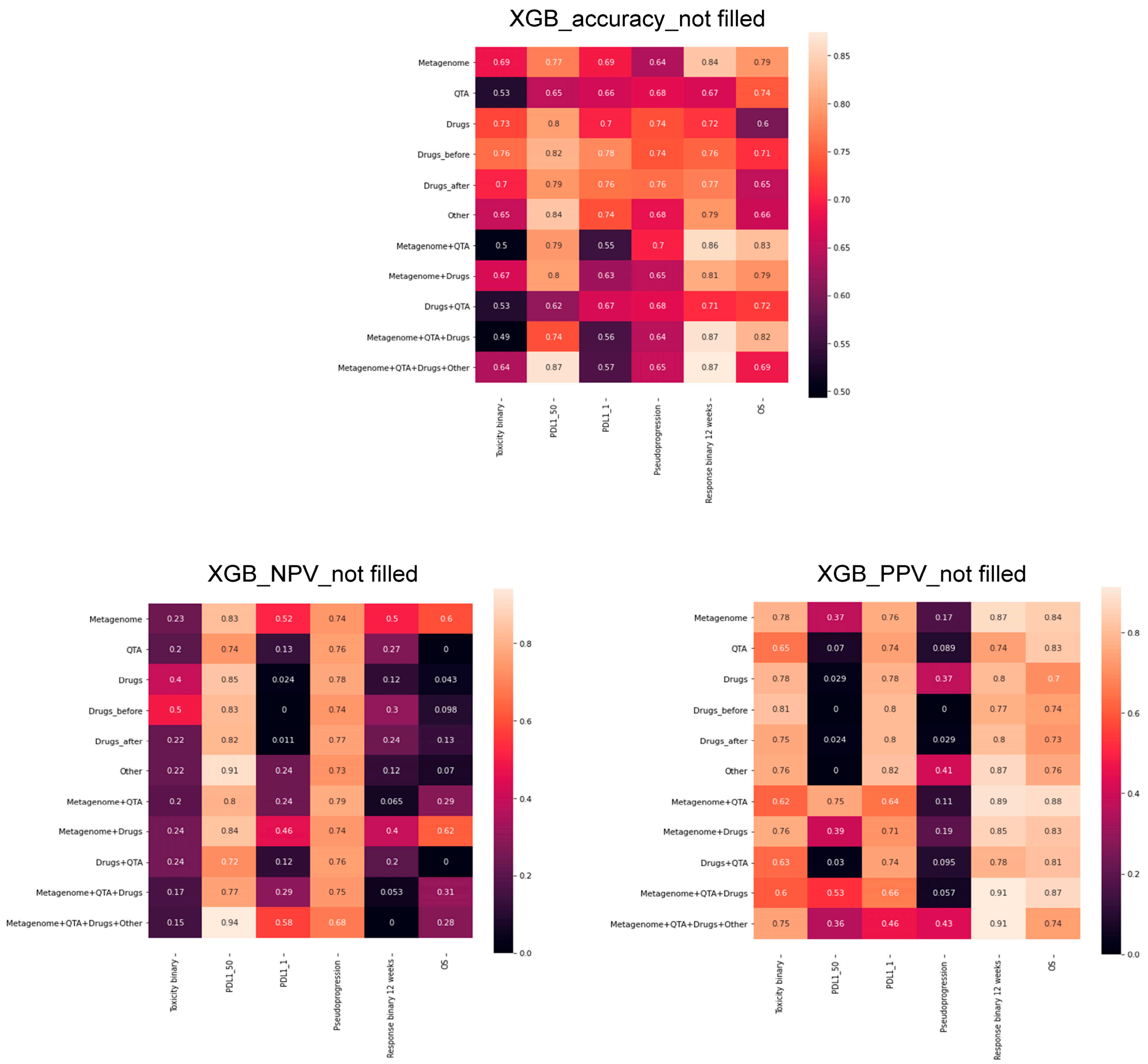

Supplementary Figure S2: Accuracy, PPV and NPV of the XGB machine learning algorithm used in predicting outcomes according to clinicopathological signatures of patients: not-filled data.

Table S1. Most abundant OTUs in Long vs Short OS patients

OS group	Kingdom	Taxon level	Taxon	Log Fc	p-value
Long OS	bacteria	speces	Parabacteroides distasonis	3.56	0.00699
Long OS	bacteria	species	Bacteroides dorei	1.11	0.00851
Long OS	fungi	species	Pezoloma ericae	0.12	0.00849
Short OS	bacteria	species	Clostridium perfringens	-3.43	0.00525
Short OS	bacteria	species	Enterococcus faecium	-1.72	0.00015
Short OS	bacteria	species	Clostridium celatum	-1.32	0.00367
Short OS	fungi	species	Cortinarius davemallochii	-11.73	0.00262
Short OS	fungi	class	Tremellomycetes unclassified	-9.3	0.00618
Short Os	fungi	species	Helotiales	-7.61	0.00304
Short OS	fungi	species	Chaetosphaeriales	-7.22	0.00154
Short OS	fungi	order	Trechisporales unclassified	-6.11	0.00155
Short OS	fungi	family	Thelephoraceae unclassified	-5.74	0.00355
Short OS	fungi	genus	Hyphodiscus unclassified	-5.11	0.00134
Short OS	fungi	genus	Coniochaeta unclassified	-4.5	0.00726
Short OS	fungi	species	Arachnopeziza aurelia	-4.44	0.00143

Short OS	fungi	species	Serendipitaceae	-4.06	0.0064
Short OS	fungi	species	Clavaria argillacea	-4.06	0.00048
Short OS	fungi	family	Lichtheimiaceae unclassified	-2.74	0.0049
Short OS	fungi	species	Meliniomyces variabilis	-2.23	0.00412
Short OS	fungi	order	Helotiales unclassified	-1.43	0.00884
Short OS	fungi	species	Acephala	-1.27	0.00853
Short OS	fungi	species	Sebacinaceae	-0.42	0.00456

Table S2. Most abundant OTUs in PD-L1-high vs PD-L1-low patients

PD-L1 group	Kingdom	Taxon level	Taxon	Log FC	p-value
High	bacteria	species	Enterococcus avium	4.35	0.00325
High	bacteria	species	Streptococcus tigurinus	2.78	0.00613
High	fungi	species	Hymenoscyphus immutabilis	7.56	0.00154
High	fungi	species	Clavulinopsis fusiformis	6.25	0.00898
High	fungi	family	Montagnulaceae unclassified	4.44	0.00384
Low	bacteria	species	Lachnospiraceae bacterium	-0.31	0.00134
Low	bacteria	species	Dorea formicigenerans	-2.59	0.00029

Table S3. Most abundant OTUs in Toxicity vs No-Toxicity patients

Toxicity group	Kingdom	Taxon level	Taxon	Log FC	p-value
Toxicity	bacteria	species	Lachnospiraceae bacterium	4.84	0.00691
Toxicity	fungi	family	Thelephoraceae unclassified	8.48	0.03463
No-toxicity	bacteria	species	Subdoligranulum	-2.71	0.03722
No-toxicity	bacteria	species	Pyramidobacter piscolens	-3.78	0.03132
No-toxicity	bacteria	species	Streptococcus lutetiensis	-7.13	0.01127
No-toxicity	fungi	species	Amanita altipes	-2.32	0.02023
No-toxicity	fungi	genus	Psathyrella unclassified	-2.58	0.02175
No-toxicity	fungi	species	Debaryomyces vindobonensis	-2.95	0.00779
No-toxicity	fungi	species	Rozellomycota	-5.36	0.02083
No-toxicity	fungi	species	Cutaneotrichosporon cutaneum	-5.93	0.01314

Table S4: Spearman's correlation coefficients and p-values of key microbial taxa, clinicopathological parameters and PCs from QTA analyses *(attached as excel)*

Table S5: 105 QTA parameters and their contribution to the 7 PCs generated by PCA. *(attached as excel)*
